# Cluster of Differentiation Markers and Human Leukocyte Antigen Expression in Chronic Lymphocytic Leukemia Patients: Correlations and Clinical Relevance

**DOI:** 10.3390/cimb46090598

**Published:** 2024-09-11

**Authors:** Maria Tizu, Bogdan Calenic, Alexandra-Elena Constantinescu, Alexandru Adrian Bratei, Razvan Antonio Stoia, Mihnea Catalin-Gabriel Popa, Ileana Constantinescu

**Affiliations:** 1 Immunology and Transplant Immunology, Carol Davila University of Medicine and Pharmacy, 258 Fundeni Avenue, 022328 Bucharest, Romania; maria.tizu@drd.umfcd.ro (M.T.); alexandra-elena.constantinescu0720@stud.umfcd.ro (A.-E.C.); mihnea-catalin-gabriel.popa0721@stud.umfcd.ro (M.C.-G.P.); ileana.constantinescu@imunogenetica.ro (I.C.); 2 Centre of Immunogenetics and Virology, Fundeni Clinical Institute, 258 Fundeni Avenue, 022328 Bucharest, Romania; 3 Academy of Romanian Scientists (AOSR), 3 Ilfov Street, Sector 5, 022328 Bucharest, Romania; 4 “Emil Palade” Centre of Excellence for Initiating Young People in Scientific Research, 3 Ilfov Street, Sector 5, 022328 Bucharest, Romania; 5 Clinical Nephrology Hospital “Carol Davila”, Calea Griviței 4, 010731 Bucharest, Romania; brateialexandru7@gmail.com; 6 Hematology Center, Fundeni Institute, 258 Fundeni Avenue, 022328 Bucharest, Romania; razvan_stoia@yahoo.com

**Keywords:** chronic lymphocytic leukemia, cluster of differentiation, human leukocyte antigen, HLA, CD, NGS, CLL

## Abstract

Chronic lymphocytic leukemia (CLL) is a distinct category of lymphoproliferative disorder characterized by the clonal expansion of mature B cells, followed by their accumulation in primary and secondary lymphoid organs. Cluster of differentiation (CD) markers such as CD79b, CD45, CD23, CD22 and CD81 serve as reliable prognostic indicators in CLL as well as the human leukocyte antigen (HLA) with its well-documented associations with various cancers. This study aims to investigate, for the first time, potential connections between HLA typing and CD marker expression in CLL. Although it is one of the most prevalent neoplasms, there is a need for biomarkers that can improve survival. This study included 66 CLL patients and 100 controls, with all samples analyzed using biochemical methods, flow cytometry, and cytomorphology. Next-generation sequencing was performed for HLA typing. The results indicate that several CD markers are statistically associated with different HLA alleles, specifically CD45 with HLA-C*07:01:01; CD79b with HLA-DPA1*02:01:02; CD23 with HLA-B*39:01:01; CD22 with HLA-B*49:01:01, HLA-C*07:01:01, HLA-DPB1*02:01:02, and HLA-DRB1*07:01:01; and CD81 with HLA-DPB1*04:02:01, HLA-DQA1*01:04:01, and HLA-DQB1*05:03:01. In conclusion, this research demonstrates significant statistical links between HLA genes and immunophenotypic markers in CLL patients, shedding new light on the immunological context of CLL.

## 1. Introduction

Lymphoproliferative disorders (LPDs) comprise a vast spectrum of maladies exhibiting diverse clinical presentations, primarily distinguished by the unrestrained proliferation of monoclonal lymphoid cells, specific characteristics that were inherited by the chronic lymphoproliferative group [1]. Generally, these disorders are characterized by an increased proliferation of lymphocytes, coupled with the absence of terminal deoxynucleotidyl transferase [2].

Among these disorders, chronic lymphocytic leukemia (CLL) is a distinctive category of LPDs which has been prominently documented over recent decades, as it stands as one of the prevailing neoplasms in developed regions [3]. Characterized by the clonal expansion of mature B lymphocytes, CLL’s initiation and development is underlined by the accumulation of these aberrant cells within the bone marrow, lymph nodes and spleen [4].

Compared to other hematological malignancies, patients with CLL have a more favorable prognosis for progression-free survival and overall survival [5]. However, the clinical presentation of CLL is complex and varied, complicating the prediction of survival outcomes due to the heterogeneous nature of the disease [5,]. To address this challenge, significant efforts have been made to improve prognostication in CLL. Two staging systems, the Rai and Binet systems, are commonly used to evaluate disease progression [6,7]. Both staging systems consider serological parameters, including lymphocyte count, platelet count and hemoglobin levels, as well as clinical indicators like the presence of adenopathies or enlargement of the spleen or liver [8]. In the Binet staging system, stages A and B are defined by the absence of anemia (hemoglobin ≥ 10 g/dL) and a platelet count ≥100,000/mm^3^, while stage C is marked by a collapse of cell lines and damage to multiple lymphoid areas [9]. Similarly, in the Rai staging system, stages I and II are characterized by a hemoglobin level ≥ 11 g/dL, a platelet count ≥ 100,000/mm^3^, and an elevated lymphocyte count. In stages III and IV, the lymphocyte count remains elevated, but platelet and/or hemoglobin levels drop below normal thresholds [8].

To enhance comprehension of the disease and improve survival prediction, various prognostic factors have been considered, including elevated β2 microglobulin (β2M) and thymidine kinase, chromosome abnormalities (17p deletion, 11q deletion, trisomy 12, unmutated immunoglobulin heavy chain variable gene (IGHV), and ZAP-70 (zeta-associated protein of 70 kDa) expression, all of which have been identified and characterized as reliable indicators of poor prognosis [7,10]. These cluster of differentiation (CD) markers also serve as important prognostic factors in LPDs in general and in CLL in particular. Thus, markers such as CD38, CD49d, CD20 and CD23 are associated with disease progression and overall survival; high expression of CD38 and CD49d often indicates a more aggressive form of CLL; CD20 and CD23 are used to help diagnose and monitor CLL, with their expression levels providing additional prognostic information [11]. These markers aid clinicians in tailoring treatment strategies and predicting patient outcomes. However, to the best of our knowledge, no studies have assessed the possible correlations between CD expression and HLA typing in CLL patients.

The human leukocyte antigen (HLA) system assumes a essential role in immune surveillance, and variations in HLA polymorphisms can influence the immune system’s capacity to recognize malignant cells and facilitate their targeted elimination [12]. The primary function of the HLA genes is to produce MHC (major histocompatibility complex) molecules responsible for identifying and presenting the antigens [13]. B cells play a crucial role in the adaptive immune response, as they can directly engage with antigens and activate T lymphocytes through the MHC molecules present on their surface [13].

It is well documented (including by our team) that HLA genes can influence the outcome of various diseases [14,15,16,17,18], including CLL [19,20,21,22]. In a recent study [23], the authors analyzed the expression patterns of HLA in CLL patients and identified that particular HLA genotypes correlate with the disease prognosis.

This study aims to investigate the potential correlations between CD marker expression and HLA typing in CLL patients. To the best of our knowledge, no previous research has explored this relationship, which could provide new insights into the prognostic significance of these biomarkers in CLL.

## 2. Materials and Methods

### 2.1. Patients and Controls

This research was carried out at the Hematology Clinic of Fundeni Clinical Institute, Bucharest, Romania, which is affiliated with “Carol Davila” University of Medicine and Pharmacy Bucharest, Romania. The study involved patients diagnosed with CLL from 2022 to 2023, and written consent was obtained from both the patient and control groups, in accordance with the Declaration of Helsinki. Ethical approval for the study was granted by the Ethical Committee of Fundeni Clinical Institute (Approval Number: 41066/29 August 2022).

To ensure consistency, the academic group involved in the study extracted, processed, and statistically analyzed medical data from each patient’s file. Patients were included in the study based on their diagnosis of CLL, and the following eligibility criteria were required: age 18 years or older, ability to provide informed consent, and absence of chromosomal disorders typically associated with CLL (del(13q), trisomy 12, del(11q), del(17p), and del(6q)). Additionally, patients were excluded if they were pregnant, had active infections, severe autoimmune diseases, or other associated cancers or diseases with a prognosis of less than 5 years, or mental disorders that could impede study participation. Patients with incomplete medical histories were also excluded. Applying all criteria resulted in the inclusion of 66 out of the initial 98 CLL-diagnosed patients. The control group consisted of 100 blood donor volunteers registered in the National Registry of Voluntary Hematopoietic Stem Cell Donors, Bucharest, Romania.

The final CLL cohort comprised 28 females and 38 males aged between 41 and 89, with a median age of 63.2 years, which was lower than the average age for these patients (approximately 70 years old) [24]. All patients had been diagnosed according to European guidelines for CLL prior to the study [8,25,]. To prevent bias, controls in the study were unrelated to the patients in the study group. Key variables such as sex and age can be found in Table 1.

### 2.2. Sample Collection and Analysis

#### 2.2.1. Cytomorphological Analysis

For the cytomorphological analysis, each study participant provided a 5 mL sample of whole blood, which was collected in Ethylene Diamine Tetra-Acetic Acid (K3EDTA) tubes (Euromed, Otopeni, Romania). These samples were used to conduct a comprehensive full blood count (FBC) using the SYSMEX XN-2000 analyzer. The FBC included measurements of hemoglobin, lymphocyte count and percentage, neutrophil count and percentage, eosinophil count and percentage, thrombocyte (platelet) count, and erythrocyte (red blood cell) count. This analysis allowed for detailed examination of the participants’ hematological profiles, which is essential for accurate diagnosis and monitoring of CLL.

#### 2.2.2. Biochemistry Markers Analysis

To determine C-reactive protein (CRP) levels, serological analysis was performed using the ABBOTT ALINITY analyzer. For this test, 5 mL of whole blood was collected from each study participant in plain vacutainers without anticoagulant. Similarly, to measure lactate dehydrogenase (LDH) levels, analysis was conducted using the SIEMENS ATELLICA CH analyzer. Again, each study participant provided a 5 mL sample of whole blood, and these samples were collected in plain vacutainers without anticoagulant. These procedures ensured the accurate measurement of both CRP and LDH values, which are important markers in the assessment of CLL.

#### 2.2.3. Flow Cytometry

Immunophenotypic analysis was conducted using the NAVIOS flow cytometer (Beckman Coulte, Brea, CA, USA) with data analyzed via Kaluza software (Version 2.1.2). Peripheral blood samples were collected in K3EDTA tubes, and 100 μL of whole blood was prepared by lysing red blood cells. The following markers were assessed: CD79b, CD20, CD43, CD38, CD11c, FMC7, CD200, kappa and lambda light chains, CD45, CD5, CD22, CD19, CD23, and CD Monoclonal antibodies conjugated with fluorochromes were added, and samples were incubated in the dark for 15–30 min. After washing with PBS, samples were analyzed on the flow cytometer and calibrated daily with standardized beads. At least 10,000 events per sample were collected. Data analysis involved gating on FSC and SSC to exclude debris, followed by specific gating for lymphocyte populations. The percentage of cells expressing each marker and the mean fluorescence intensity (MFI) were quantified. Isotype and fluorescence-minus-one (FMO) controls ensured accuracy and reproducibility. This analysis provided detailed immunophenotypic profiles for accurate CLL classification and prognostication.

#### 2.2.4. HLA Analysis

For HLA genotyping, 5 mL of whole blood per individual was collected from both patients and controls using EDTA tubes. DNA extraction was carried out using the QIAmp DNA Blood Mini^®^ kit (QIAGEN, Hilden, Germany). Acceptable samples exhibited an A260 nm/A280 nm ratio between 1.7 and 1.9, indicating solution purity, and a DNA concentration exceeding 20 ng/µL. The extracted DNA underwent sequencing to identify HLA gene polymorphisms using next-generation sequencing (NGS). The MIA FORA NGS MFlex HLA protocol (MIA FORA™ NGS MFlex) was as detailed in our previous study on HLA-CLL associations [23]. Subsequently, the NGS library, prepared with Illumina reagents, was loaded onto an Illumina MiniSeq sequencer (Illumina, San Diego, CA, USA). Post-sequencing, data analysis was performed using the MIA FORA NGS FLEX software V2.1.1 (Sirona Genomics, Inc., Portland, OR, USA), alongside two reference databases: IMGT and Sirona Genomics databases (V3.49.0).

### 2.3. Statistical Analysis

Statistical analysis was performed using the chi-square test or Fisher’s exact test. Odds ratios (ORs) with 95% confidence intervals (CIs) were calculated to determine the strength of associations. Statistical significance was set at a regression analysis *p* < 0.05. SPSS V28.0 was used for the statistical analysis of the results.

## 3. Results

In this study, a series of hematological and biochemical parameters were determined for the cohort of CLL patients, along with CD marker expression and HLA genotyping. The values obtained for hematological and biochemical parameters were compared to reference intervals, and the percentages of patients with values outside these intervals are presented in Table 2.

For the selected patients, the immunophenotype was determined using 20 markers. Their expression levels were classified as positive, weak positive, or negative. The number and percentage of patients in each category are presented in Table 3.

Following analysis, the hematological and biochemical markers and the specific CD markers, we searched for correlations between these two types of parameters. Our findings indicated that the white blood cells count (WBC) is significantly correlated to CD38 expression (*p* = 0.0193) as CD38 positivity is more frequent in patients with WBC > 55,000 µL^−1^ (OR = 4.1, lower 95% CI = 1.0145, upper 95% CI = 16.5697). Other slight correlations were with CD81 expression (*p* = 0.1144) and with CD79b expression (*p* = 0.1965) as CD81 positivity is more frequent in patients with WBC < 18,500 µL^−1^ (OR = 2.75, lower 95% CI = 0.9139, upper 95% CI = 8.275) and CD79b is more frequent positive in patients with WBC > 27,000 µL^−1^ (OR = 2.23, lower 95% CI = 0.8194, upper 95% CI = 6.08).

Lymphocytes number is also significantly correlated to CD38 expression (*p* = 0.0087) and CD38 positivity occurs more frequently in patients with lymphocytes number > 45,000 µL^−1^ (OR = 7, lower 95% CI = 1.52, upper 95% CI = 32.24). Another slight correlation regards CD22 positivity (*p* = 0.171) as it occurs more frequently in patients having less than 6500 lymphocytes per µL (OR = 4.05, lower 95% CI = 1.24, upper 95% CI = 13.21).

Regarding neutrophils number, no significant correlation has been observed, but some observations have been performed as further described. Positive staining is more frequent for CD81 in patients who have more than 4500 neutrophils per µL (OR = 2.5, lower 95% CI = 0.818, upper 95% CI = 7.642) and for CD38 in patients who have more than 4400 neutrophils per µL (OR = 5.95, lower 95% CI = 1.125, upper 95% CI = 31.473).

For eosinophils number, a significant correlation was identified, with CD79b positivity (*p* = 0.039) defined as 66.66% of the patients having intense positivity greater than 200 eosinophils per µL.

No significant correlation has been obtained with the LDH levels, but some observations have been recorded. It was observed that all the CD23-negative cases were described as patients with LDH > 470 U/L. Another observation was that intense CD43 positivity is more frequent in patients who have LDH levels over 360 U/L (OR = 3.48, lower 95% CI = 0.848, upper 95% CI = 14.281).

CRP levels were strongly correlated with CD5 positivity (*p* = 0.02) and slightly correlated with CD22 positivity (*p* = 0.074). Intense positivity for CD5 was more frequent in patients with CRP < 5 mg/L (OR = 2.235, lower 95% CI = 0.82, upper 95% CI = 6.09) and 70% of the patients with negative CD5 staining-associated CRP levels > 5 mg/L. On the other hand, CD22 positivity was observed more frequently in patients who had CRP > 5 mg/L (OR = 4.643, lower 95% CI = 1.338, upper 95% CI = 16.106). Besides these associations, the HLA expression and the molecular phenotype were studied to determine any correlations and a series of observations were conducted. The *p*-values determined using regression analysis are given in Table 4 below. Each biomarker’s expression has been classified as absent, weak or strong and correlated with HLA relevant for CLL (Table 5).

Starting from the correlations listed above, some observations were made. Regarding CD20, it was observed that its positivity is related to the presence of HLA-DRB1*11:04:01 and HLA-B*49:01:01, as 50% of the patients with strong CD20 expression and 17.5% of the patients with weak CD20 expression showed an association with at least one of the two HLAs, while none of the patients with absent CD20 expression presented any of the two HLAs. On the other hand, HLA DRB1*15:02:01 is associated with a diminished expression of CD20, as none of the patients with a strong CD20 expression showed an association with it and 12.5% of the patients with no CD20 or a weak CD20 expression showed an association with the given HLA.

CD45 expression is positively correlated with HLA-B*18:01:01, HLA-C*07:01:01, HLA-DQB1*02:01:01 and HLA-DQA1*05:01:01, as 57.89% of the patients with a strong CD45 expression showed an association with at least one of the four HLAs, while the patients with absent CD45 expression showed no association with any of the above-mentioned HLAs. On the other hand, CD45 expression is negatively correlated with HLA-B*35:01:01, HLA-DRB1*11:01:01, HLA-DRB1*01:01:01 and HLA-DQA1*01:02:02, as 71.43% of the patients with absent CD45 expression showed an association with at least one of the four HLAs compared to only 31.58% of the patients with strong CD45 expression.

For CD43 expression, three HLAs were found to be positively correlated, while two other HLAs were found to be negatively correlated with the expression of CDBy. Upon calculating the difference between the number of positively correlated HLAs and negative correlated HLAs, it was observed that the difference was at least one for 58.33% of the strongly CD43-positive patients, while 79.63% of the CD43-negative or weakly positive patients associated a difference equal to zero or less.

The expression of CD79b is positively correlated with HLA-DPA1*02:01:02 and HLA-B*08:01:01, as 57.14% of the strongly CD79b-positive patients showed an association with at least one of the two HLAs, while 86.44% of the CD79b-negative or weakly positive patients did not show any association with either of the two listed HLAs.

By using the four HLAs that were positively correlated with CD22 expression (HLA-B*49:01:01, HLA-C*07:01:01, HLA-DPB1*02:01:02 and HLA-DRB1*11:01:01), it was observed that 85.714% of the patients with strong CD22 expression showed an association with at least two out of the four HLAs, while 88.14% of the patients with weak or no CD22 expression showed an association with no more than one out of the four positively correlated HLAs.

Regarding CD81 expression, it was observed that 57.14% of the patients with weak or no CD81 expression showed an association with at least one of the two negatively correlated HLAs (HLA-DPB1*04:02:01 and HLA-DRB4*01:03:01), whereas none of the patients with strong CD81 expression showed such an association. For complete data supporting the persented results please check Supplementary Materials found in Tables S1–S3.

## 4. Discussion

The present study analyzed CD markers and their possible relationship with HLA expression to enhance the understanding of chronic lymphocytic leukemia (CLL) and improve survival prediction. CD markers such as CDCD22, CD23, CDCD45, and CDCD81 are particularly noteworthy for their roles in disease progression and prognosis. By investigating the expression patterns of these CD markers in conjunction with HLA genotyping, we aimed to identify potential correlations that could offer deeper insights into the disease mechanisms and patient outcomes. Understanding these relationships is crucial for developing more accurate prognostic tools and personalized treatment strategies. The main CD markers involved in CLL are presented in Table 6, providing an overview of their significance in the context of this study.

While CD22 is commonly associated with acute lymphoblastic leukemia [54], it is also present in various other hematological disorders like CLL, especially in atypical forms of CLL [42]. Its occurrence in CLL typically indicates significant impairment of secondary lymphoid tissue, frequently leading to the presence of lymphadenopathy and splenomegaly in affected patients [41]. Within our cohort, we observed a statistically significant correlation between CD22 and the HLA-B*49:01:01, HLA-C*07:01:01, HLA-DPB1*02:01:02 and HLA-DRB1*07:01:01 alleles. Among these alleles, the existing literature highlights associations between aplastic anemia and HLA-B*49:01:01 [55], acute myeloid leukemia and HLA-C*07 [56] and childhood common acute lymphoblastic leukemia and HLA-DPB1*02:01 [57]. Regarding the involvement of HLA-DRB1*07:01:01 in the occurrence of CLL, the study conducted by Aung et al. [58] concludes that HLA-DRB1*07 is more involved in the occurrence of follicular lymphoma and does not increase the risk of CLL. These associations between CD22 positivity and HLA alleles indirectly suggest a higher specificity for acute leukemias, despite their potential identification in other pathological conditions. It should be noted that the presence of strong CD22, which is an indicator for atypical forms, is associated with alleles (HLA-B*49:01:01, HLA-C*07:01:01 and HLA-DPB1*02:01: 01) generally found in other malignancies, suggesting that this association may be representative for patients with atypical CLL. This discovery is accompanied by an observed association with the absence of CD22, with the presence of HLA-DRB1*07:01:01 identified as having a protective role in patients with CLL, indicating a protective role of these two markers in these patients.

One of the distinguishing features of leukocytes is the presence of CD45, also referred to as lymphocyte common antigen [59]. CD45 is present in both T and B lymphocytes and functions as a receptor-linked protein tyrosine phosphatase, playing a role in cellular activation and signaling within these cells [60,61]. Detection of CD45 can be achieved through techniques like immunohistochemistry or flow cytometry [62,63]. Its examination is essential in confirming the hematopoietic origin of a tumor. Notably, high levels of CD45 expression may indicate a poorer prognosis in specific hematologic malignancies, such as pediatric B cell progenitor acute lymphoblastic leukemia [64,65] and multiple myeloma [66]. Research on CD45 expression in chronic lymphoproliferative diseases, particularly in B cell chronic lymphocytic leukemia (B-CLL), has been conducted extensively [31,67]. Multiple research teams have found that CD45 serves as a valuable marker for distinguishing between typical and atypical forms of CLL [31]. Usually, the presence of weakly positive CD45 is associated with the typical form of CLL, whereas atypical forms exhibit brighter expression of CD45, indicating its utility in diagnostic differentiation [31,68]. Our research has identified significant statistical correlations between CD45 and HLA-C*07:01:01, HLA-DQA1*05:01:01, and HLA-DQA1*01:02:01. While HLA-DQA1*05:01:01 is predominantly recognized for its involvement in the development of Celiac disease [14,69,], previous studies, such as the one conducted by Gragert et al. [19] have highlighted the importance of HLA-C*07:01 in CLL, while our team mentioned and HLA-DQA1*01:02:01 for its tendency to approach the limit of statistical significance in CLL [23]. Therefore, certain HLA genes linked to CD45 play a crucial role in the development of CLL, highlighting the significance of CD45 as a valuable marker for improving our understanding of CLL. Describing an atypical picture for CLL, we have identified the presence of strong CD45 expression in association with HLA-C*07:01, also mentioned for its association with CD22 in patients with atypical forms of CLL. This leads us to the idea that we have identified another important characteristic of patients with atypical forms of CLL.

Together with CD19, CD22, and CD79b, CD20 stands out as a surface marker of B cells [70]. It holds particular significance among surface antigens, with extensive research paving the way for the development of targeted antibody therapies (anti-CD20 antibody, also known as Rituximab) [71,72,73]. These therapies are now utilized in the treatment of non-Hodgkin B cell lymphoma, autoimmune diseases, ABO incompatible transplantation, and transplant rejection [71,72,74,75]. While CD20 is present in 40% of pre-B acute lymphoblastic leukemia/lymphoblastic lymphoma cases, it is also observed in diffuse large B cell lymphomas (DLBCLs) and large B cell lymphomas, as well as CLL [73,76,77]. Notably, in CLL, CD20 tends to be negative or significantly reduced in most cases [77], consistent with our findings that characterize CD20 as weakly positive for the majority of our patients. In our study group, we noted a statistically significant correlation between CD22 and HLA-B*49:01:01, HLA-DRB1*11:04:01, HLA-DRB1*15:02:01 and HLA-DPA1*01:03. While the literature lacks extensive documentation on the relationship between HLA-B*49:01:01 and CLL, as noted in the context of its association with CD22, this particular allele appears to serve as a promising marker for aplastic anemia [55]. As for the role of HLA-DRB1*11:04:01 in disease occurrence, evidence suggests its implication in acute lymphoblastic leukemia (ALL) [78] and hairy cell leukemia, with hemolytic uremic syndrome [79]. Notably, a study by Morsi et al. [80] highlights that the combined involvement of HLA-DRB1*11 and HLA-DRB1*15 alleles in predicting an unfavorable response to therapy in acute and chronic myeloid leukemia. From this viewpoint, while the link between CD22 and CLL is evident, the identified HLA genes were not previously associated with CLL occurrence. Instead, they appear to be implicated in the development of acute leukemia. Although we would expect CD20 to be negative for our patients, some of them presented strong CD20, which was associated with HLA-B*49:01:01 and HLA-DRB1*11:04:01. The presence of this strong CD20 is atypical, while HLA-B*49:01:01 was mentioned earlier because it is associated with CD22 in atypical forms of CLL, and HLA-DRB1*11:04:01, although nonspecific for CLL, is associated with the occurrence of complications. Thus, we can conclude that the association of these markers is unfavorable for patients with CLL.

While CD43 is recognized as a surface marker predominantly found in T lymphocytes [81], it has also been detected in various malignant cells, including those associated with CLL, as well as in myeloid malignancies [82,83,84]. Taking into account the study conducted by Falay et al. [37], which suggests that CD43 is predominantly a positive marker for atypical forms of CLL, our observation of generally absent or weakly positive CD43 expression in our patient group suggests that they predominantly exhibit typical forms of CLL. Additionally, our research team uncovered a notable association between HLA-DRB1*15:01:01 and CD43 in the limited number of CD43-positive patient group, hinting at a potential immunological profile characteristic of atypical CLL forms. Unfortunately, the literature regarding the role of HLA-DRB1*15:01:01 in CLL occurrence is not sufficiently extended and only mentions the involvement of this allele in myeloid leukemias [80].

CD23 serves as a marker for B cells, notably in diseases such as SLL (Small Lymphocytic Lymphoma)/CLL, diffuse follicular lymphoma, and mediastinal large B cell lymphoma [85]. Its presence aids in distinguishing SLL/CLL (CD23+) from mantle cell lymphoma or MALT lymphoma (CD23-) [86]. Regarding the involvement of HLA-B*39:01:01 and HLA-A*11:01:01 in CLL development, research indicates that HLA-B*39:01:01 is one of the alleles previously identified by our team for its association with CLL susceptibility in women [23]. Also, in a different study, our team established a significant association between HLA-A*11:01 and peripheral T-cell lymphoma not otherwise specified (PTCL-NOS) [18]. Despite 80% of our patient cohort displaying the CD23 marker, the association with HLA-B*39:01:01 and HLA-A*11:01:01, typically linked to CLL patients, was unexpected. Specifically, these alleles were significantly associated only in patients with negative or weak CD23 expression, suggesting the presence of an atypical form of disease, in which the patients do not present CD23 but have one of the mentioned alleles and present LDH values > 470 U/L, which attests to the gravity factor.

Recognized as a marker of B lymphocytes, CD79 holds significance in its role within the composition of the antigen receptor complex of B lymphocytes. It plays a crucial role in the maturation and activation of these lymphocytes. CD79 consists of CD79a subunits, which are present in the cytoplasm of immature lymphocytes, and CD79b subunits, found in mature lymphocytes. CD79b has also notably been identified as an important marker for CLL [85], although there are studies that stipulate that only a percentage of patients with CLL express CD79b [87]. In our patient cohort, subjects lacking CD79b expression exhibited a correlation with HLA-A*32:01:01, whereas patients with CD79b expression also displayed an association with HLA-DPA1*02:01:02. Although these alleles were not previously linked to CLL or other leukemias, they contribute to the overall immunological profile of these patients.

Another significant finding was that heightened expression of CD79b correlated with an elevated white cell count (WBC > 27,000) and correlated directly with an increased number of eosinophils in affected patients. The predominantly weak positive expression of CD79b is also documented in the literature concerning patients with CLL. Conversely, high CD79b expression predicts an unfavorable outcome in CLL [40] and can also suggest potential complications such as Richter syndrome [88], explaining the high white cell count and eosinophils count encountered in these patients. The association between HLA-DPA1*02:01:02 and bright CD79b in patients with WBC over 2700/L and eosinophils over 200/L indicates a serious picture of an atypical form of the disease, with an unfavorable prognosis.

Additionally, studies like the one conducted by Schlette E. et al. [87] discuss the simultaneous presence of CD22 with CD79b, in a similar manner to what was observed in our investigation. Also, McCarron K. F. et al. [89] reported that combining CD79b determination alongside CD5 was of huge importance in resolving misclassification, making both essential to use in testing panels for CLL. In our patient cohort, both CD5 and CD22 markers proved to be significant, with the majority of patients showing positive or weakly positive expression. Notably, both markers exhibited a strong correlation with CRP levels. CD5 positivity was linked to CRP values < 5 mg/L, whereas CD22 positivity was more frequently observed in patients with CRP levels > 5 mg/L.

CD81 stands out as a significant marker in characterizing hematological malignancies [52,90]. It can be detected in lymphocytes as well as myeloid cells, playing a crucial role in distinguishing between lymphoid and myeloid disorders and also between different types of LPDs [52,90]. Typically, CLL exhibits either negative CD81 expression or weak presence [51,90], results that align with the findings of our team. Studies emphasize CD81’s diagnostic role in ALM alongside its potential in therapeutic applications through anti-CD81 monoclonal antibodies [91,92,93]. Furthermore, researchers have documented the significance of CD81 as a marker in the diagnosis of MCL [52]. As a member of the tetraspanin family, CD81 exhibits a significant correlation with both MHC class I and class II [94,95]. In our population, we have observed a connection between CD81 and the following HLA class II genes: HLA-DPB1*04:02:01, HLA-DQA1*01:04:01, HLA-DQB1*05:03:01, and HLA-DRB1*14:01:01. Although the literature has not yet documented the involvement of HLA-DRB1*14 in CLL, a study by Zhou et al. suggests its implication in ALL [96]. Similarly, there is evidence indicating the involvement of HLA-DQ5 (corresponding to the extended molecular typing HLA-DQB1 *0501-*0504) in both adult and pediatric cases of ALL [97]. Nonetheless, the literature also discusses the protective role of HLA-DQB1*05 against Hodgkin lymphoma [98], complemented by the protective effect conferred by HLA-DPB1*04:01 in this disease [99]. However, it is also important to note that CLL cells lacking this marker exhibit a relationship that approaches statistical significance concerning white cell count (WBC reaching up to 18,500/L). The fact that the HLA-DRB1*14:01:01, HLA-DQB1*05:03:01 and HLA-DQA1*01:04:01 alleles are associated with a strong expression of CD81, which is not exactly specific for CLL, indicates a form of severity, especially in the context of WBC values up to 18,500/L.

In accordance with our findings, other studies have identified CD38 as being one of the most important markers when it comes to evaluating CLL, as its presence is highly associated with an aggressive clinical course [100,101]. Previously correlated with a high incidence of lymph node involvement, anemia, hepatomegaly, and elevated β2M levels, CD38 expression stands out as a significant indicator of risk [102]. Numerous studies have associated its presence with the aggressive progression of the disease because in CLL, a proliferating tumor cell demonstrates elevated levels of CD38 expression [103,104,105], while others have linked it to early-stage disease (Rai stages 0-II) [102]. Nonetheless, its presence undoubtedly serves as a warning sign for reduced survival and increased treatment requirements [101,106]. Our data suggest a concerning clinical scenario among CD38+ patients with a white blood cell count exceeding 55,000 (*p* = 0.0193, OR = 4.1) and lymphocyte counts surpassing 45,000 µL (*p* = 0.0087, OR = 7), raising concerns about an abrupt evolution of the disease and making it necessary to administer the medication much faster.

Although our research cannot currently change the management of patients with CLL, we believe that we can draw some important conclusions that can represent the basis of future studies. Our results show that several of the HLA-CD associations are related to atypical forms of CLL (strong CD22 is associated with HLA-B*49:01:01, HLA-C*07:01:01 and HLA-DPB1*02:01:01, strong CD45 is associated with HLA-C*07:01, strong CD20 is associated with HLA-B*49:01:01 and HLA-DRB1*11:04:01, strong CD79b is linked with HLA-DPA1*02:01:02, and there is an association between HLA-DRB1*14:01:01, HLA-DQB1*05:03:01 and HLA-DQA1*01:04:01 with bright CD81). Since the presence of these atypical markers was accompanied by important changes in hematological markers (WBC over 2700/L in the case of patients with CD79b and WBC values up to 18,500 for patients with bright CD81) and biochemical markers (strong CD23 associated with LDH over 470 U/L) we conclude that patients in whom these atypical correlations are present face an unfavorable evolution with reserved prognosis. This raises the issue of special management of these atypical forms. The mentioned HLA-CD associations are clear markers of the presence of atypical forms, and thus, the evolution of these patients could be followed up and future studies should take into account the time needed to initiate the treatment and the patients’ response to the treatment, whether we are talking about classical treatment or the necessary new forms of treatment.

While our study uncovers new and intriguing correlations among CLL, immunophenotypic markers and HLA alleles, it is important to acknowledge that it has several limitations. An important aspect is the fact that we could not prove for certain that the HLA-CD association offers a clearly superior result in determining the severity of the disease, especially in the case of atypical forms of CLL. Thus, it is possible that the expression of the CD marker alone provides more information about the patient’s condition than the HLA-CD association. However, this aspect can only be clarified through future studies, for which the present study is the starting point. Besides that, another constraint is the small patient cohort size, attributed to the relative rarity of CLL and the specific inclusion/exclusion criteria used. Additionally, the intrinsic polymorphism of HLA genes presents a challenge in establishing definitive associations between these genes and particular diseases. Future studies should improve matching between controls and patients with regard to gender and age variables.

## 5. Conclusions

Our preliminary results indicate that CD20 is correlated with HLA-DRB1*11:04:01, HLA-DRB1*15:02:01 and HLA-B*49:01:01 alleles; CD45 is correlated with HLA-C*07:01:01, HLA-DQA1*05:01:01 and HLA-DQA1*01:02:02; CD79b is correlated with HLA-DPA1*02:01:02 and HLA-A* 32:01:01; CD23 is correlated with HLA-B*39:01:01 and HLA-A* 11:01:01; CD43 is correlated with HLA-DRB1*15:01:01; CD22 is correlated with HLA-B*49:01:01, HLA-C*07:01:01, HLA-DPB1*02:01:02 and HLA-DRB1*07:01:01; and CD81 is correlated with HLA-DPB1*04:02:01, HLA-DQA1*01:04:01, HLA-DQB1*05:03:01 and HLA-DRB1*14:01:01 in Romanian patients with CLL. The field of HLA and disease association represents a promising area of research, expanding our understanding of the genetic determinants influencing immunity. In summary, this study highlights clear statistical associations between HLA genes and immunophenotypic markers in CLL patients, providing deeper insights into the immunological background of CLL.

## Figures and Tables

**Table 1:** General characteristics of cases and controls.

		Gender		Age (Years)
	Total	Male (%)	Female (%)	Mean
CLL patients *	66	57.5%	42.4%	63.2
Controls	100	55%	45%	34.3

* CLL: chronic lymphocytic leukemia.

**Table 2:** Values of the selected biochemical and hematological parameters.

Parameter	Determined Levels	Reference Interval	% of Patients Outside the Reference Interval
WBC * (10^3^ µL^−1^)	11.31–89.05	3.98–10	87.88%
Hemoglobin (g × dL^−1^)	11.39 ± 2.66	11.2–17.5	39.39%
Lymphocytes (10^3^ µL^−1^)	4.58–50.55	1.8–3.74	92.06%
% Lymphocytes	64.09 ± 25.5	19.3–53.1	79.37%
Neutrophils (10^3^ µL^−1^)	1.99–5.39	1.56–6.13	34.92%
% Neutrophils	3.7–32.9	34–71.1	85.71%
Eosinophils (10^3^ µL^−1^)	0.065–0.3	0.04–0.54	28.57%
% Eosinophils	0.1–1.3	0.7–7	61.29%
Thrombocytes (10^3^ µL^−1^)	149.72 ± 77.18	150–450	53.85%
Erithrocytes (10^3^ µL^−1^)	3.86 ± 1.03	3.93–6.08	43.08%
LDH (U/L)	373.02 ± 111.53	208–378	46.97%
CRP (mg/L)	2.5–7.5	0–3	66.67%

* WBC stands for white blood cells count.

**Table 3:** Immunophenotype—statistically relevant CDs.

Marker	Number of Positive Patients	Percent of Positive Patients	Number of Weak Positive Patients	Percent of Weak Positive Patients	Number of Negative Patients	Percent of Negative Patients
CD20	18	27.27%	40	60.61%	8	12.12%
CD45	57	86.36%	2	3.03%	7	10.61%
CD43	12	18.18%	19	28.79%	35	53.03%
CD79b	7	10.61%	22	33.33%	37	56.06%
CD5	29	43.94%	26	39.39%	10	15.15%
CD22	7	10.61%	12	18.18%	47	71.21%
CD23	53	80.30%	7	10.61%	6	9.09%
CD81	3	4.55%	16	24.24%	47	71.21%

**Table 4:** Statistical correlations between HLA expression and relevant CD markers (*p*-value).

Biomarker	HLA	*p*-Value
CD20	HLA-DRB1*11:04:01	0.028521
	HLA-DRB1*15:02:01	0.041458
	HLA-DPA1*01:03:01	0.046239
	HLA-B*49:01:01	0.043283
CD45	HLA-C*07:01:01	0.047395
	HLA-DQA1*05:01:01	0.032716
	HLA-DQA1*01:02:02	0.01544
CD79b	HLA-DPA1*02:01:02	0.024732
	HLA-A*32:01:01	0.024347
CD23	HLA-B*39:01:01	0.007212
	HLA-A* 11:01:01	0.017837
CD43	HLA-DRB1*15:01:01	0.038263
CD22	HLA-B*49:01:01	0.000472
	HLA-C*07:01:01	0.025517
	HLA-DPB1*02:01:02	0.038567
	HLA-DRB1*07:01:01	0.02458
CD81	HLA-DPB1*04:02:01	0.002678
	HLA-DQA1*01:04:01	0.000192
	HLA-DQB1*05:03:01	0.000621
	HLA-DRB1*14:01:01	0.000065

* Statistical significance was determined after calculating the *p*-value.

**Table 5:** Analysis for the selected biomarkers and HLAs.

Biomarker	HLA	Correlation	HLA Presence	OR	Sup 95% CI	Inf 95% CI
CD20	HLA-DRB1*11:04:01	Positive	33.33% of the patients with strong CD20 expression and 12.5% of the patients with weak or no CD20 expression	3.5	12.853	0.953
	HLA-DRB1*15:02:01	Negative	25% of the patients with no CD20 expression and 6.9% of the patients with weak or strong CD20 expression	4.5	0.676	29.948
	HLA-B*49:01:01	Positive	16.66% of the patients with strong CD20 expression and 2.08% of the patients with weak or no CD20 expression	9.4	0.909	97.26
CD45	HLA-C*07:01:01	Postive	Only in patients with strong CD45 expression; in 33.33% of the patients	-	-	
	HLA-DQA1*05:01:01	Postive	Only in patients with strong CD45 expression; in 36.84% of the patients	-	-	-
	HLA-DQA1*01:02:02	Negative	28.57% of the patients with no CD45 expression; 6.78% of the patients with weak or strong expression	5.5	37.84	0.8
CD79b	HLA-DPA1*02:01:02	Positive	42.86% of the patients with strong CD79b expression and 3.39% of the patients with weak or absent CD79b expression	21.375	167.12	2.73
	HLA-A* 32:01:01	Negative	Only in patients with no CD79b expression; in 18.92% of the patients with negative CD79b	-	-	-
CD23	HLA-B*39:01:01	Negative	23.08% of the patients with weak or no CD23 expression and 3.77% of the patients with strong CD23 expression	7.65	51.83	1.13
	HLA-A* 11:01:01	Negative	15.39% of the patients with weak or no CD23 expression and 3.77% of the patients with strong CD23 expression	4.64	36.58	0.59
CD43	HLA-DRB1*15:01:01	Positive	41.66% of the patients with strong CD43 expression and 11.11% of the patients with weak or absent CD43 expression	5.71	23.82	1.37
CD22	HLA-B*49:01:01	Positive	42.86% of the patients with strong CD22 expression and 1.7% of the patients with weak or absent CD22 expression	43.5	519.27	3.64
	HLA-C*07:01:01	Positive	42.11% of the patients with strong or weak CD22 expression and 23.4% of the patients with no CD22 expression	2.38	7.4	0.766
	HLA-DPB1*02:01:02	Positive	71.43% of the patients with strong CD22 expression and 28.81% of the patients with weak or absent CD22 expression	6.18	34.98	1.09
	HLA-DRB1*07:01:01	Negative	Only in patients with no CD22 expression; in 28.81% of the patients with negative CD22	-	-	-
CD81	HLA-DPB1*04:02:01	Negative	44.68% of the patients with no CD81 expression and 5.26% of the patients with weak or strong CD81 expression	14.54	118.04	1.79
	HLA-DQA1*01:04:01	Positive	21.05% of the patients with strong or weak CD81 expression and 2.13% of the patients with no CD81 expression	12.27	118.44	1.16
	HLA-DQB1*05:03:01	Positive	26.32% of the patients with strong or weak CD81 expression and 4.26% of the patients with no CD81 expression	8.04	46.06	1.4
	HLA-DRB1*14:01:01	Positive	26.32% of the patients with strong or weak CD81 expression and 2.13% of the patients with no CD81 expression	16.43	152.61	1.77

* Statistical significance was determined after calculating OR (odds ratio), and CI (confidence interval).

**Table 6:** CD marker expression in chronic lymphocytic leukemia.

Marker	Role	References
CD20	CD20 is a biomarker for normal and neoplastic and mature and immature B cells	[26]
	CD20+ cells are usually associated with the cytotoxic CD8+ category, followed by the helper CD4+ compartment	[27]
	In healthy individuals, CD20+ cell counts are significantly higher when compared to those observed in patients with CLL	[28]
	Utilizing engineered or alternative anti-CD20 monoclonal antibodies (mAbs) could enhance the efficacy of immunotherapy for chronic lymphocytic leukemia	[29]
	Survival rates in acute lymphoblastic leukemia (ALL) are higher in patients without CD20 expression (CD20−) compared to those with CD20 expression (CD20+)	[30]
CD45	CD45 is a specific marker for CLL and plays a critical role in the diagnosis and classification of this disease	[31]
	CD45 is employed in differential diagnosis to distinguish between typical CLL and non-CLL B cell Chronic Lymphoproliferative Disorders (CLPDs)	[32]
	Its expression is critical for identifying and categorizing lymphocyte subpopulations within hematological conditions, thereby facilitating a more precise clinical assessment and enabling targeted therapeutic strategies	[33]
	CD45 expression demonstrates statistically significant variations across different ethnic groups; therefore, it could be utilized for diagnostic purposes and disease categorization in the fields of public health science and geoepidemiology	[34]
CD43	The majority of circulating B cells do not express CD43, except for a small subset of activated B cells. Additionally, CD43 expression is noted at varying levels across a spectrum of B cell lymphomas.	[35]
		[36]
	CD43 can be considered as definitive markers in atypical CLL patients with potential for use in the differential diagnosis of typical/atypical CLL	[37]
		[38]
CD79	CD79 is a critical component of the B cell receptor complex, playing key roles in B cell activation, development, cell survival and homeostatis	[39] [40]
	CD79 is a valuable marker for monitoring minimal residual disease (MRD) in patients with CLL. As a marker, it enables the detection of persistent CLL cells post-treatment, aiding in the evaluation of therapeutic efficacy and the prediction of potential disease recurrence	
	Elevated CD79 expression is associated with poor prognosis in CLL and may also indicate the potential for complications	
CD22	CD22 is usually expressed on mature B cells and less on immature B cells	[41] [42] [43] [44] [45] [46]
	CD22 is involved in B cell survival, development and homeostatis	
	Monoclonal antibodies that target CD22 have been developed to target and deplete malignant/autoreactive B cells	
	Altered expression of CD22 affects the progression and treatment of CLL	
	Individuals with CLL that express lymphadenopathy and/or splenomegaly tend to be CD22+	
	CD22 is more positively correlated with accute lymphoblastic leukemia than chronic lymphoblastic leukemia	
	Due to its critical roles in modulating B cell receptor signaling, CD22 presents a promising therapeutic target for immunosuppressive interventions in future clinical applications	
CD23	CD23 is mainly expressed in B lymphocytes and is actively involved in mediating allergic reactions through regulation of IgE production	[47]
	High levels of CD 23 expression are common in CLL and are used to differentiate chronic lymphocytic leukemia from other B cell malignancies	[48]
	Blood-soluble levels of CD23 serve as a prognostic marker in CLL	[49]
	CD23 is a potential therapeutic target with several mAb designed to block its activity, leading to better disease control	[50]
CD81	CD81 is a transmembrane protein expressed by cells of the immune system, including B and T cells	[38] [51] [52] [53]
	Is involved in B cell activation and proliferation and in T cell adhesion and signaling	
	CD81 expression on leukemia cells acts as a diagnostic marker for specific types of leukemia	
	It alters the interaction between leukemia cells and T cells and may impair the response of the immune system to leukemia	
	It may represent therapeutic targets with mAb acting as inhibitors of CD81 and enhancing the clearance of leukemia cells.	

## Data Availability

The data that support the findings of this study are available on request.

## Supplementary Materials

The following supporting information can be downloaded at https://www.mdpi.com/article/10.3390/cimb46090598/s1, Table S1. The selected immunophenotyping markers—all CDs tested; Table S2. Statistical correlations between HLA expression and CD markers; Table S3. Analysis for the selected biomarkers and HLAs.
