# Supplementary material for: Cluster of Differentiation Markers and Human Leukocyte Antigen Expression in Chronic Lymphocytic Leukemia Patients: Correlations and Clinical Relevance
Source: Curr Issues Mol Biol. 2024 Sep 11;46(9):10008–25. doi: 10.3390/cimb46090598 (PMC11430089; doi:10.3390/cimb46090598)
Supplement: Supplementary file 1 [file cimb-46-00598-s001.zip › cimb-3158203-supplementary.pdf]

Supplemental Table S1. The selected immunophenotyping markers – all CDs tested.

Marker	Number of positive patients	Percent of positive patients	Number of weak positive patients	Percent of weak positive patients	Number of negative patients	Percent of negative patients
**CD19**	66	100.00%	0	0.00%	0	0.00%
**CD 10**	0	0.00%	0	0.00%	66	100.00%
**CD20**	18	27.27%	40	60.61%	8	12.12%
**CD45**	57	86.36%	2	3.03%	7	10.61%
**CD 43**	12	18.18%	19	28.79%	35	53.03%
**CD79b**	7	10.61%	22	33.33%	37	56.06%
**CD5**	29	43.94%	26	39.39%	10	15.15%
**CD 8**	0	0.00%	0	0.00%	66	100.00%
**CD22**	7	10.61%	12	18.18%	47	71.21%
**CD23**	53	80.30%	7	10.61%	6	9.09%
**CD 25**	1	1.52%	0	0.00%	65	98.48%
**CD200**	56	84.85%	4	6.06%	6	9.09%
**CD11c**	0	0.00%	3	4.55%	63	95.45%
**CD81**	3	4.55%	16	24.24%	47	71.21%
**CD31**	0	0.00%	1	1.52%	65	98.48%
**CD38**	1	1.52%	9	13.64%	56	84.85%
**CD 56**	0	0.00%	0	0.00%	66	100.00%
**CD 103**	1	1.52%	0	0.00%	65	98.48%
**Kappa chains**	42	63.64%	0	0.00%	24	36.36%
**Lambda chains**	24	36.36%	0	0.00%	42	63.64%

Supplemental Table S2. Statistical correlations between HLA expression and CD markers.

Biomarker	HLA	p-value	Biomarker	HLA	p-value
CD20	HLA-DRB1*11:04:01	0.028521	CD43	HLA-C*04:01:01	0.096629
	HLA-DRB1*15:02:01	0.041458		HLA-C*12:03:01	0.059286
	HLA-DPA1*01:03:01	0.046239		HLA-DQA1*03:01:01	0.069925
	HLA-B*49:01:01	0.043283		HLA-DQB1*06:01:01	0.099848
CD45	HLA-B*18:01:01	0.092181		HLA-DRB1*15:01:01	0.038263
	HLA-C*07:01:01	0.047395	CD22	HLA-B*49:01:01	0.000472
	HLA-DQB1*02:01:01	0.078698		HLA-C*07:01:01	0.025517
	HLA-DQA1*05:01:01	0.032716		HLA-DPB1*02:01:02	0.038567
	HLA-B*35:01:01	0.052071		HLA-DRB1*07:01:01	0.02458
	HLA-DRB1*11:01:01	0.082376		HLA-DRB1*11:01:01	0.073203
	HLA-DRB1*01:01:01	0.08558		HLA-DRB4*01:01:01	0.059597
	HLA-DQA1*01:02:02	0.01544	CD81	HLA-DPB1*04:02:01	0.002678
CD79b	HLA-DPA1*02:01:02	0.024732		HLA-DQA1*01:04:01	0.000192
	HLA-DQA1*01:03:01	0.082654		HLA-DQB1*05:03:01	0.000621
	HLA-B* 08:01:01	0.10525		HLA-DRB1*14:01:01	6.59E-05
	32:01:01HLA	0.024347		HLA-DRB4*01:03:01	0.060266
CD5	HLA-DPB1*03:01:01	0.07186	Kappa and Lambda chains	HLA-B*35:01:01	0.061339
	HLA-DRB1*13:03:01	0.095337		HLA-DQA1*01:03:01	0.061339
CD23	HLA-B*39:01:01	0.007212		HLA-DQB1*02:02:01	0.061339
	11:01:01HLA	0.017837		HLA-DRB1*15:02:01	0.053232

Supplemental Table S3. Analysis for the selected biomarkers and HLAs.

Biomarker	HLA	Correlation	HLA presence	OR	sup 95% CI	inf 95% CI
CD20	HLA-DRB1*11:04:01	Positive	33.33% of the patients with strong CD20 expression and 12.5% of the patients with weak or no CD20 expression	3.5	12.853	0.953
	HLA-DRB1*15:02:01	Negative	25% of the patients with no CD20 expression and 6.9% of the patients with weak or strong CD20 expression	4.5	0.676	29.948
	HLA-B*49:01:01	Positive	16.66% of the patients with strong CD20 expression and 2.08% of the patients with weak or no CD20 expression	9.4	0.909	97.26
CD45	HLA-B*18:01:01	Postive	Only in patients with strong CD45 expression; in 26.32% of the patients	-	-	-
	HLA-C*07:01:01	Postive	Only in patients with strong CD45 expression; in 33.33% of the patients	-	-	-

	HLA-DQB1*02:01:01	Positive	Only in patients with strong CD45 expression; in 29.07% of the patients	-	-	-
	HLA-DQA1*05:01:01	Positive	Only in patients with strong CD45 expression; in 36.84% of the patients	-	-	-
	HLA-B*35:01:01	Negative	42.86% of the patients with no CD45 expression; 11.86% of the patients with weak or strong expression	5.57	30.26	1.03
	HLA-DRB1*11:01:01	Negative	42.86% of the patients with no CD45 expression; 13.56% of the patients with weak or strong expression	4.78	25.456	0.898
	HLA-DRB1*01:01:01	Negative	28.57% of the patients with no CD45 expression; 6.78% of the patients with weak or strong expression	5.5	37.84	0.8
	HLA-DQA1*01:02:02	Negative	28.57% of the patients with no CD45 expression; 6.78% of the patients with weak or strong expression	5.5	37.84	0.8
**CD43**	HLA-C*04:01:01	Negative	38.89% of the patients with weak or no CD43 expression and 16.66% of the patients with strong CD43 expression	3.18	15.98	0.63
	HLA-C*12:03:01	Positive	50% of the patients with strong CD43 expression and 22.22% of the patients with weak or absent CD43 expression	3.5	12.85	0.66
	HLA-DQA1*03:01:01	Negative	31.43% of the patients with no CD43 expression and 12.9% of the patients with weak or strong CD43 expression	3.09	11.01	0.87
	HLA-DQB1*06:01:01	Positive	25% of the patients with strong CD43 expression and 3.7% of the patients with weak or absent CD43 expression	8.67	59.36	1.27
	HLA-DRB1*15:01:01	Positive	41.66% of the patients with strong CD43 expression and 11.11% of the patients with weak or absent CD43 expression	5.71	23.82	1.37
**CD79b**	HLA-DPA1*02:01:02	Positive	42.86% of the patients with strong CD79b expression and 3.39% of the patients with weak or absent CD79b expression	21.375	167.12	2.73
	HLA-DQA1*01:03:01	Negative	21.62% of the patients with no CD79b expression and 6.9% of the patients with weak or strong CD79b expression	3.72	19.12	0.73
	HLA-B*08:01:01	Positive	42.86% of the patients with strong CD79b expression and 10.17% of the patients with weak or absent CD79b expression	6.625	36.95	1.19
	HLA-A*32:01:01	Negative	Only in patients with no CD79b expression; in 18.92% of the patients with negative CD79b	-	-	-
**CD5**	HLA-DPB1*03:01:01	Positive	27.59% of the patients with strong CD5 expression and 8.11% of the patients with weak or absent CD5 expression	4.32	18.12	1.03
	HLA-DRB1*13:03:01	Positive	13.79% of the patients with strong CD5 expression and 2.7% of the patients with weak or absent CD5 expression	5.76	54.65	0.61
**CD22**	HLA-B*49:01:01	Positive	42.86% of the patients with strong CD22 expression and 1.7% of the patients with weak or absent CD22 expression	43.5	519.27	3.64
	HLA-C*07:01:01	Positive	42.11% of the patients with strong or weak CD22 expression and 23.4% of the patients with no CD22 expression	2.38	7.4	0.766

	HLA-DPB1*02:01:02	Positive	71.43% of the patients with strong CD22 expression and 28.81% of the patients with weak or absent CD22 expression	6.18	34.98	1.09
	HLA-DRB1*07:01:01	Negative	Only in patients with no CD22 expression; in 28.81% of the patients with negative CD22	-	-	-
	HLA-DRB1*11:01:01	Positive	31.58% of the patients with weak or strong CD22 expression and 10.64% of the patients with no CD22 expression	3.88	14.81	1.02
	HLA-DRB4*01:01:01	Negative	Only in patients with no CD22 expression; in 19.15% of the patients with negative CD22	-	-	-
**CD23**	HLA-B*39:01:01	Negative	23.08% of the patients with weak or no CD23 expression and 3.77% of the patients with strong CD23 expression	7.65	51.83	1.13
	HLA-A* 11:01:01	Negative	15.39% of the patients with weak or no CD23 expression and 3.77% of the patients with strong CD23 expression	4.64	36.58	0.59
**CD81**	HLA-DPB1*04:02:01	Negative	44.68% of the patients with no CD81 expression and 5.26% of the patients with weak or strong CD81 expression	14.54	118.04	1.79
	HLA-DQA1*01:04:01	Positive	21.05% of the patients with strong or weak CD81 expression and 2.13% of the patients with no CD81 expression	12.27	118.44	1.16
	HLA-DQB1*05:03:01	Positive	26.32% of the patients with strong or weak CD81 expression and 4.26% of the patients with no CD81 expression	8.04	46.06	1.4
	HLA-DRB1*14:01:01	Positive	26.32% of the patients with strong or weak CD81 expression and 2.13% of the patients with no CD81 expression	16.43	152.61	1.77
	HLA-DRB4*01:03:01	Negative	38.3% of the patients with no CD81 expression and 15.79% of the patients with weak or strong CD81 expression	3.31	12.98	0.84
**Kappa chains**	HLA-B*35:01:01	Positive	21.43% of the patients with kappa chains expression and 4.17% of the patients with lambda chains expression	6.27	52.97	0.74
	HLA-DQA1*01:03:01	Positive	21.43% of the patients with kappa chains expression and 4.17% of the patients with lambda chains expression	6.27	52.97	0.74
	HLA-DQB1*02:02:01	Positive	21.43% of the patients with kappa chains expression and 4.17% of the patients with lambda chains expression	6.27	52.97	0.74
	HLA-DRB1*15:02:01	Positive	Only in patients with kappa chains expression; in 14.29% of the patients with positive kappa chains expression	-	-	-
